# Probiotics Prevents Sensitization to Oral Antigen and Subsequent Increases in Intestinal Tight Junction Permeability in Juvenile–Young Adult Rats

**DOI:** 10.3390/microorganisms7100463

**Published:** 2019-10-16

**Authors:** Janyerkye Tulyeu, Hideki Kumagai, Eriko Jimbo, Shinya Watanabe, Koji Yokoyama, Longzhu Cui, Hitoshi Osaka, Makiko Mieno, Takanori Yamagata

**Affiliations:** 1 Department of Pediatrics, Jichi Medical University, 3311-1 Yakushiji, Shimotsuke, Tochigi 3290498, Japan; janerket@gmail.com (J.T.); erikojimbo@jichi.ac.jp (E.J.); kokuresyuke@jichi.ac.jp (K.Y.); hosaka@jichi.ac.jp (H.O.); takanori@jichi.ac.jp (T.Y.); 2 Department of Immunology and Laboratory, School of Biomedicine, Mongolian National University of Medical Sciences, Jamyan St 3, Ulaanbaatar 14210, Mongolia; 3 Division of Bacteriology, Department of Infection and Immunity, Jichi Medical University, 3311-1 Yakushiji, Shimotsuke, Tochigi 3290498, Japan; swatanabe@jichi.ac.jp (S.W.); longzhu@jichi.ac.jp (L.C.); 4 Department of Medical Informatics, Center for Information, Jichi Medical University, 3311-1 Yakushiji, Shimotsuke, Tochigi 3290498, Japan; mnaka@jichi.ac.jp

**Keywords:** barrier, claudin, food allergy, occludin, ovalbumin, probiotics, sensitization, zonula occludens

## Abstract

Increased intestinal permeability is thought to underlie the pathogenesis of food allergy. We explore the mechanism responsible for changes in the morphology and function of the intestinal barrier using a rat model of food allergy, focusing on the contribution of intestinal microbiota. Juvenile–young adult rats were sensitized with ovalbumin and treated with antibiotics or probiotics (*Clostridium butyricum* and *Lactobacillus reuteri*), respectively. The serum ovalbumin-IgE levels, intestinal permeability, histopathological features, tight junction (TJ)-associated proteins, Th2 cytokines, and gut microbiota in feces were analyzed in each group. Sensitized rats showed an increase in ovalbumin-IgE levels and intestinal permeability with gut mucosal inflammation, whereas rats that received probiotics were only mildly affected. Rats given ovalbumin, but not those given probiotics, showed a reduction in both TJ-related protein expression and localization. Th2 cytokine levels were increased in the sensitized rats, but not in those given probiotics. TJs in rats treated with ovalbumin and antibiotics were disrupted, but those in rats administered probiotics were undamaged. Clostridiaceae were increased in the probiotics groups, especially *Alkaliphilus*, relative to the ovalbumin-sensitized group. Gut microbiota appears to play a role in regulating epithelial barrier function, and probiotics may help to prevent food sensitization through the up-regulation of TJ proteins.

## 1. Introduction

Food allergy, the pathogenesis of which is still unknown, has become a serious public health issue, as its prevalence has increased over the last two decades and around 10% of children are affected [1]. Probiotics are live microorganisms that confer a health benefit on the host when administered in adequate amounts [2]. Currently, the Food Allergy and Anaphylaxis Guidelines of the European Academy of Allergy and Clinical Immunology state “there is no evidence to recommend prebiotics or probiotics or other dietary supplements based on particular nutrients to prevent food allergy” [3]. The number of reports on both basic experiments and clinical trials designed to clarify the effects of probiotics or gut microbiota on allergic disorders has been increasing [4,5,6,7,8,9,10,11,12]. For instance, *Clostridium butyricum* significantly ameliorates intestinal anaphylaxis symptoms in mice with food allergy [13]. Pre- and postnatal *Lactobacillus reuteri* supplementation decreases allergen responsiveness in infancy [14]. Although these probiotics (*C. butyricum* and *L. reuteri*) have been widely used for their expected health benefits, evidence for their effectiveness against food allergy is still insufficient.

The intestinal barrier, which acts as both a mechanical and a microbial barrier, plays an important role in the development of immune tolerance to prevent allergens passing through the intestinal epithelia from the external environment [15,16]. Thus, increased intestinal permeability is thought to be associated with the pathogenesis of food allergy. Recently, it has been reported that the intestinal microbiota contributes to the organization of epithelial barrier function, and changes the bacterial community linked to intestinal permeability and chronic gastrointestinal disease, especially food allergy [17,18].

The aim of the present study was to explore the mechanism responsible for changes in the morphology and function of the intestinal barrier using a juvenile–young adult rat model of food sensitization, focusing on the contribution of intestinal microbiota and the effects of probiotics (*C. butyricum* and *L. reuteri*) and antibiotics. In this context, previous fragmentary reports have indicated that in ovalbumin (OVA)-sensitized rats, intestinal permeability is increased in association with damage to mucosal tight junctions (TJs) and expression of molecules involved in TJ regulation [19], or that probiotics decrease Th2 responses and improve intestinal barrier function [20]. However, the mechanisms underlying the relationship between intestinal epithelial barrier function and regulation of gut microbiota in the context of food allergy have not been precisely clarified, and further accumulation of scientific data is needed. With this aim, we have been studying the use of different types of probiotics and antibiotic treatments in OVA-sensitized rats. Here, we conducted a comprehensive investigation of serum-IgE, gut permeability, ultrastructural features, previously unreported TJ-associated proteins (e.g., claudin-1, -3, -5, and -7), Th2 cytokines, and fecal microbiota, and evaluated the relationships among them. As the results, the TJs of OVA-sensitized rats were disrupted, whereas rats that received probiotics were only mildly affected. The sensitized rats also showed a reduction in both TJ-related proteins expression and localization. Clostridiaceae were increased by probiotics administration, especially *Alkaliphilus*, relative to the ovalbumin-sensitized group. Thus, gut microbiota appears to play a role in regulating TJ proteins leading to barrier function.

## 2. Materials and Methods

### 2.1. Animal Handling and Study Design

**Figure 1:**
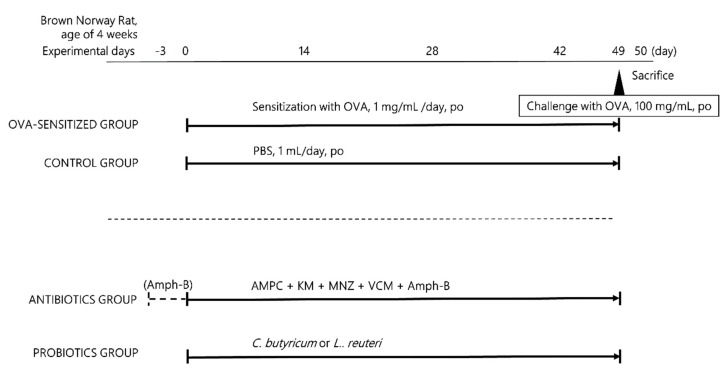
Protocol used for ovalbumin (OVA) sensitization, antibiotic or probiotic treatment, or challenge. Brown Norway rats in the control (PBS only) group were sensitized with PBS (1 mL per rat, daily) intragastrically for 48 days: in the OVA-treated group, rats were sensitized with OVA (1 mg in 1 mL PBS, daily per rat) intragastrically for 48 days. Both groups were subdivided into those receiving added antibiotics (amphotericin-B, ampicillin, kanamycin, metronidazole, and vancomycin) or probiotics (*Clostridium butyricum* and *Lactobacillus reuteri*). All OVA-sensitized rats received challenges on day 49 with 100 mg/mL OVA (in PBS 1 mL), and all rats in those groups were sacrificed and blood was collected on day 50 for further study. AMPC; ampicillin, Amph-B; amphotericin-B, KM; kanamycin, MNZ; metronidazole, OVA; ovalbumin, VCM; vancomycin.

Four-week-old male Brown Norway SPF rats were obtained from Charles River Laboratories (Tokyo, Japan). They were housed under specific pathogen-free conditions at 23 (±3) °C with a 12 h light/dark cycle and a relative humidity of 30%–70% during experiments, and were provided with conventional food and water ad libitum. Protocols for all animal studies were approved by the Institutional Animal Experiment Committee of Jichi Medical University (Approval Number: 17082-01), in accordance with the Institutional Guidelines for Proper Conduct of Animal Experiments and Related Activities in Academic Research Institutions under the jurisdiction of the Ministry of Education, Culture, Sports, Science, and Technology. Rats were divided into an antigen sensitized group and a control group. In the sensitized group, 1 mg of OVA (Worthington Biochemical Corp, Lakewood, NJ, USA) in 1 mL of PBS was administrated intragastrically daily for 48 days without the use of an adjuvant, and the control group was administered 1 mL of PBS in the same way. Each group was further subdivided into one receiving antibiotics for ablation of intestinal bacteria and one receiving probiotics (*C. butyricum* and *L. reuteri*). On day 49, all OVA-sensitized rats were then orally challenged with OVA (100 mg) solution in 1 mL of PBS [19]. On day 50, the intestine and blood were collected from every rat (*n* = 5–7 rats per group: PBS only; 6, PBS + antibiotics (Abx); 5, PBS + *C. butyricum* (CB); 5, PBS + *L. reuteri* (LR); 5, OVA only; 7, OVA + Abx; 7, OVA + CB; 7, OVA + LR; 6) (Figure 1).

### 2.2. Ablation of Intestinal Flora by Antibiotic Treatment

Antibiotic treatment was performed in accordance with a previous report with some modification [21]. Briefly, amphotericin-B (Fuji Pharma, Tokyo, Japan) was administered by gavage at 1 mg/kg every 12 hours before the start of the experiment. From day zero, water flasks freely available to the rats were supplemented with 1 g/L ampicillin (Astellas Pharma, Tokyo, Japan), and an antibiotic cocktail consisting of 50 mg/kg vancomycin (Shionogi Pharma, Tokyo, Japan), 100 mg/kg kanamycin (Meiji Seika Pharma, Tokyo, Japan), 100 mg/kg metronidazole (Shionogi Pharma, Tokyo, Japan), and 1 mg/kg amphotericin-B was administered by antibiotic gavage every 12 hours. A gavage volume of 10 mL/kg body weight was delivered via a gastric tube without prior sedation. The antibiotic cocktail was prepared freshly every day, and ampicillin and water were renewed every seventh day (Figure 1).

### 2.3. Probiotic Treatment

Probiotic treatment groups comprising an antigen sensitized group and a control group were further divided into three subgroups. The probiotics (*C. butyricum* [MIYAIRI 588®, Miyarisan Pharmaceutical Co., Ltd., Tokyo, Japan] at 1 × 10^8^ CFU/mL, *L. reuteri* [DSM 17938, Bio Gaia Japan Co., Ltd., Stockholm, Sweden] at 1 × 10^9^ CFU/mL, respectively; 5–7 rats per group) were administrated respectively by daily gavage with 1 mg OVA in 1 mL PBS for seven weeks (49 days) in the sensitized group. The control group received the same concentrations and dosages of probiotics in 1 mL PBS in the same way (Figure 1).

### 2.4. Measurement of Serum OVA-IgE

Blood was collected from the jugular vein on days 0, 14, 28, and 50 from the start of the experiment. Each sample was allowed to clot for 1 h at room temperature and was then centrifuged at 2000× *g* (15 min, 4 °C); all sera were stored at −20 °C. The serum OVA-specific IgE was assayed by ELISA in accordance with the manufacturer’s instructions (Cusabio Technology LLC, Houston, TX, USA). The final OD value was detected at 450 nm wavelength using a microplate reader (Benchmark Plus, Microplate Reader, Bio-Rad, USA). We prepared a standard curve by plotting standard concentration on the X-axis and absorbance on the Y-axis, and used it to calculate the IgE concentration of each sample from its absorbance.

### 2.5. Evaluation of Intestinal Permeability 

Intestinal permeability was determined by measuring the lactulose/mannitol ratio in urine samples in each group (days 14, 28, and 50). After a 24 h fast, rats were administrated 100 mg of lactulose and 50 mg of mannitol (dissolved in 1 mL distilled water) orally. The percentage absorption of these sugars was determined from the amount of excreted lactulose and mannitol measured during the first six hours after ingestion, using an EnzyChrom intestinal permeability assay kit (BioAssay Systems, Hayward, CA, USA) in accordance with the manufacturer’s instructions. Any increase in this ratio indicated increased intestinal permeability, as lactulose is only absorbed though intercellular spaces. 

### 2.6. Hematoxylin and Eosin (HE) Staining 

On day 50, intestinal samples were collected during deep anesthesia by intraperitoneal injection of Nembutal (Dainippon Sumitomo, Tokyo, Japan). For HE staining, specimens of the jejunum were fixed with 4% paraformaldehyde in a 50 mM phosphate buffer (Wako, Osaka, Japan), pH 7.4, for 24 h at 4 °C and stained with HE after dehydration, embedding, and slicing. The structure and morphological changes were observed and analyzed using a microscope (Olympus, Tokyo, Japan). Villus length was determined by measuring the distance from the crypt base to the villus tip using ImageJ software (Version 1.50, National Institutes of Health, Bethesda, MD, USA). The degree of inflammation was evaluated using an intestinal inflammation scoring system based on the following parameters: (1) inflammatory cell infiltration, (2) damage to the surface epithelium, and (3) irregular villous and crypt loss [22]. We also counted eosinophil infiltration in the lamina propria of the jejunal mucosa. Five to seven animals from each experimental group were evaluated, and a minimum of 15 well-oriented villi from each section were measured and observed by microscopy. 

### 2.7. Transmission Electron Microscopy

Under deep anesthesia, small pieces (about 1.5 mm × 1.5 mm × 2 mm) of the jejunum were rapidly excised and fixed with 2.5% glutaraldehyde in a 0.1 M phosphate buffer, pH 7.4, for 2 h at 4 °C. The specimens were then post-fixed with 1% osmium tetroxide in a 0.1 M phosphate buffer for 1.5 h at 4 °C. The pieces of the jejunum were then dehydrated in a graded ethanol series, transferred to propylene oxide, embedded in epoxy resin (Quetol 812; Nisshin EM Co., Tokyo, Japan), and polymerized for 48 h at 60 °C. The specimen blocks were cut into ultrathin sections with an ultramicrotome (UCT; Leica Microsystems, Waltzer, Germany), stained with uranyl acetate and lead citrate, after which the structural and morphological changes in epithelial cells and tight junctions (TJs) were examined using a transmission electron microscope (HT7700; Hitachi, Tokyo, Japan). The apical junction length and width of the TJ and adherens junction of jejunum epithelial cells were measured using ImageJ software (Version 1.50, National Institutes of Health, Bethesda, MD, USA). Representative data were obtained from 10–15 measurements per sample (*n* = 5–7 per samples per group).

### 2.8. Immunofluorescence Staining

The fixed jejunal samples were immersed in 30% sucrose in 50 mM PBS for two days at 4 °C, embedded in Tissue-Tek OCT compound (Sakura FineTechnical, Tokyo, Japan), and frozen on dry ice. Cryosections (thickness: 4 μm) were obtained using a cryostat (CM3000; Leica Microsystems, Wetzlar, Germany) and blocked with 2% normal goat serum (Vector Laboratories, Burlingame, CA, USA) for 30 min at 30 °C. Tissues were incubated in antibody retrieval buffer with 0.01 M citrate, pH 6.0, for 10 min at 90 °C, and then with primary antibodies against the TJ proteins claudin-1 (Cat#717800), claudin-2 (Cat#51600), claudin-7 (Cat#349100), and zonula occludens (ZO-1) (Cat#617300) [Thermo Fisher Scientific (Waltham, MA, USA)], claudin-3 (SAB4200607), and claudin-5 (SAB4200537) [Sigma-Aldrich (St. Louis, MO, USA)] at 4 °C overnight. After being washed with PBS, the fixed jejunal tissues were incubated with the secondary antibody Alexa-Fluor-488-conjugated goat anti-rabbit IgG (Thermo Fisher Scientific, Waltham, MA, USA) for 30 min at 30 °C. Nuclei were visualized in Vectashield Hardset Mounting medium with DAPI (Vector Laboratories, CA, USA). Imaging was performed using a confocal laser microscope, FluoViewTM FV1000 (Olympus, Tokyo, Japan), equipped with ×20 and ×40 objective lenses. 

### 2.9. Real-Time PCR 

After the rats were sacrificed on day 50, 25–40 mg of jejunal tissue was collected and ground in liquid nitrogen, and then transferred to a 1.5 mL EP tube. The total RNA (*n* = 5–7 rats per group) was extracted using TRIzol Reagent (Cat#15596018, Invitrogen, Carlsbad, CA, USA) and reverse-transcribed to cDNA using a Superscript® VILO^TM^ kit (Invitrogen, Carlsbad, CA, USA). Primers for TJ proteins and cytokines were designed using the Primer-Blast software package (National Center for Biotechnology Information, Rockville, Bethesda, MD, USA) based on the mRNA sequences in GenBank (National Center for Biotechnology Information, Bethesda, MD USA). These primer-sequences are listed in Table 1. Real-time PCR was performed using ABI 7500 Fast Real-time PCR (ABI 7500; Applied Biosystems, Life Technologies, Thermo Fisher Scientific, Waltham, MA, USA) and samples were run in triplicate. The reaction conditions were as follows: 50 °C for 2 min, 95 °C for 2 min for the holding step, followed by 40 cycles of 95 °C for 3 s and 60 °C for 30 s. As an internal control, β-actin was used for standardization of the transcript results, and relative gene expression levels were calculated by the (2^−ΔΔCT^) method. We confirmed the relative quantitative PCR (2^−ΔΔCT^) values between OVA sensitization rats and non-sensitization rats. We adopted PBS only (control) samples instead of non-sensitization samples because there were no remarkable differences between them.

### 2.10. Fecal DNA Isolation and 16S rRNA Sequence Analysis

On day 50, feces were collected in a sterile tube filled with 1 mL PBS and then immediately frozen at −80 °C. For isolation of DNA, 100–300 mg of fecal material was ground with silica beads and extracted with a QIAamp DNA stool mini kit (Qiagen, Hilden, Germany) in accordance with the manufacturer’s instructions. PCR products for the V4 region of the 16S rRNA gene were amplified with region-specific primers that included the Illumina flowcell adapter (Illumina, San Diego, CA, USA) sequences and 12-base barcodes on the reverse primer. PCR production of the bacterial DNA template was quantified using Invitrogen’s PicoGreen. Taxonomic classification of 16S rRNA targeting amplicon reads and the eight most abundant bacterial sequences was performed using Illumina 16S Metagenomics workflow in the Miseq Reporter software curated by the GreenGene taxonomic database (https://basespace.illumina.com/analyses/). Alpha diversity was calculated based on the Shannon index for richness and evenness of bacterial sequences at rarefraction depth reads of the operational taxonomic unit sample. 

### 2.11. Statistical Analysis

All group comparisons, except the microbiota analysis, were performed using analysis of variance (ANOVA) with the Tukey–Kramer method for multiple comparison after normality test. The microbiota analysis was performed using permutational multivariate analysis of variance (PERMANOVA) using PRIMER version 7 software (PRIMER-e, New Zealand). Box and whisker plots were created using GraphPad Prism 7 software (GraphPad Software, USA). Differences at *p* < 0.05 were considered to be statistically significant.

## 3. Results

### 3.1. Serum OVA-IgE Level and Intestinal Permeability

**Figure 2:**
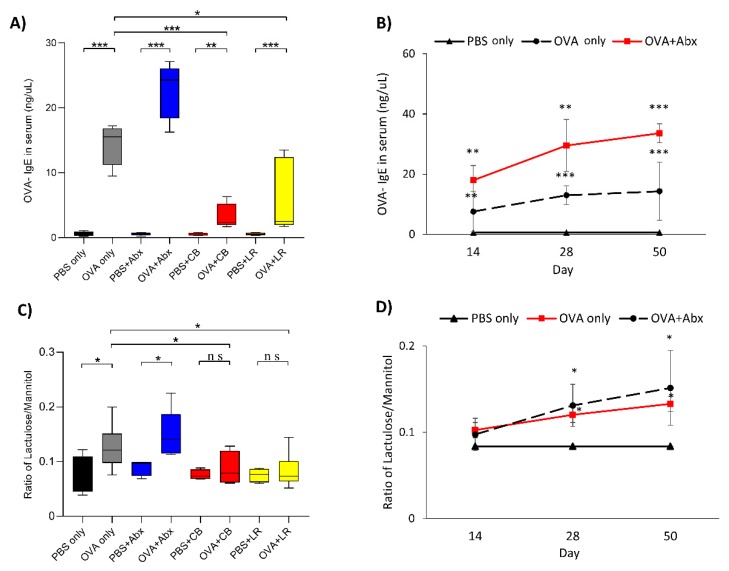
(**A**) Serum OVA-IgE level rose in all OVA-sensitized rats, but was significantly decreased by probiotics administration in comparison with the OVA sensitization group. (**B**) Time course of serum OVA-IgE level. Serum OVA-IgE level began to rise significantly from the second week in both the OVA sensitization and OVA sensitization with antibiotics groups. The levels in the OVA sensitization groups continued with no differences between those with and without antibiotic administration. (**C**) Intestinal permeability assay of lactulose/mannitol metabolism and absorption. An increase in this ratio indicates increased intestinal permeability. (**D**) Time course of intestinal permeability. The degree of intestinal permeability began to rise significantly from the fourth week in both the OVA sensitization and OVA sensitization with antibiotics groups. The intestinal permeability levels in the OVA sensitization groups continued with no differences between those with and without antibiotic administration. Boxes represent the 25th to 75th percentiles; middle bar identifies the median; whiskers show the minimum and maximum (*n* = 5–7 rats per group). * *p* < 0.05, ** *p* < 0.01, *** *p* < 0.001 determined by analysis of variance (ANOVA) with the Tukey–Kramer method. ns, no significance; Abx, antibiotics; CB, *C. butyricum*; LR, *L. reuteri*.

Food sensitization was induced successfully in OVA-sensitized rats compared with controls, as confirmed by a significant (*p* < 0.001) increase of serum-specific OVA-IgE values after seven weeks of sensitization. The group subjected to OVA sensitization with antibiotic treatment showed the highest value (Figure 2A). The value of OVA-IgE began to rise significantly from the second week in both the OVA sensitization (OVA only) and OVA sensitization with antibiotics groups. The OVA-IgE levels in the OVA sensitization groups continued with no differences between those with and without antibiotic administration (Figure 2B). In contrast, administration of probiotics led to a significant decrease in the value of IgE in the OVA sensitization groups (*p* < 0.05) (Figure 2A). An increase in the lactulose/mannitol ratio indicated that intestinal permeability was significantly increased in both the OVA sensitization (OVA only) group and the OVA sensitization with antibiotics group relative to the PBS only (control) groups (Figure 2C). The degree of intestinal permeability began to rise significantly from the fourth week in both the OVA sensitization (OVA only) and OVA sensitization with antibiotics groups. The intestinal permeability levels in the OVA sensitization groups continued with no differences between those with and without antibiotic administration (Figure 2D). In contrast, groups subjected to OVA sensitization followed by treatment with each of the probiotics showed a significant decrease of the lactulose/mannitol ratio in comparison with the OVA sensitization alone group (Figure 2D). 

### 3.2. Histological Examinations of the Jejunal Tissue

**Figure 3:**
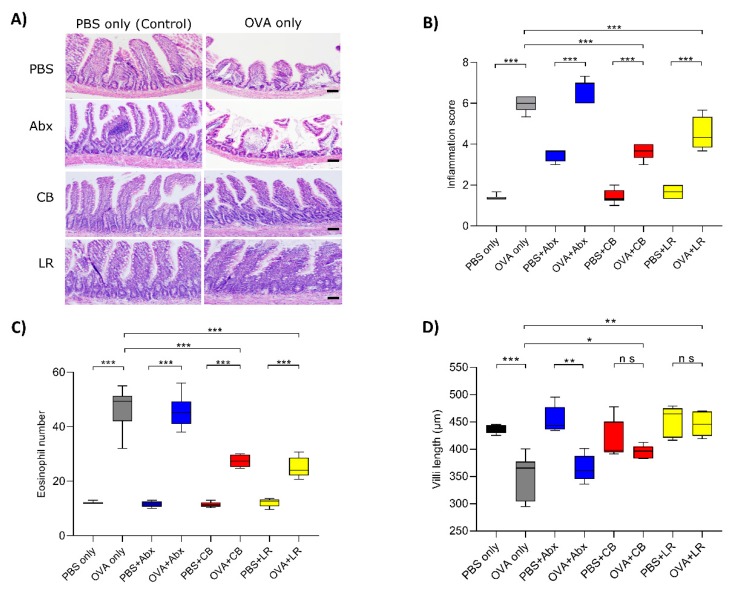
(**A**) Jejunal tissue sections were stained with hematoxylin and eosin for histological examination. Although the jejunal villi were damaged in the OVA-sensitized group and those in rats that had received antibiotics, those in rats that had received probiotics were only mildly affected. Scale bars, 100 μm. (**B**) Histological examination was performed by assigning a score for epithelial damage and leukocyte infiltration on microscopic cross-sections of the jejunum in each rat. (**C**) The number of infiltrating eosinophils was counted in the lamina propria of the jejunum, and representative numbers were obtained from 10–15 measurements per sample. (**D**) Villus length in jejunal epithelial cells was measured. Boxes represent the 25th to 75th percentiles; middle bar identifies the median; whiskers show the minimum and maximum (*n* = 5–7 rats per group). * *p* < 0.05, ** *p* < 0.01, *** *p* < 0.001 determined by ANOVA with the Tukey–Kramer method. ns, no significance; Abx, antibiotics; CB, *C. butyricum*; LR, *L. reuteri.*

HE staining showed that the jejunal mucosae were inflamed in all of the OVA sensitization groups (Figure 3A). The histological inflammation score in those groups was significantly increased (*p* < 0.001) and eosinophil infiltration was also significantly increased (*p* < 0.001) in all OVA sensitization groups relative to the control (PBS only) (Figure 3B,C). In contrast, the intestinal inflammation scores and degrees of eosinophil infiltration were significantly reduced by probiotic treatment in comparison with the OVA sensitization group (OVA only) (Figure 3B,C). Moreover, villus length in the OVA only group and OVA with antibiotics treatment group was significantly reduced (*p* < 0.01), although the probiotic treatment groups showed almost normal features similar to those in the controls (Figure 3D). 

### 3.3. Examination of Epithelial Intercellular Structures under Electron Microscopy

**Figure 4:**
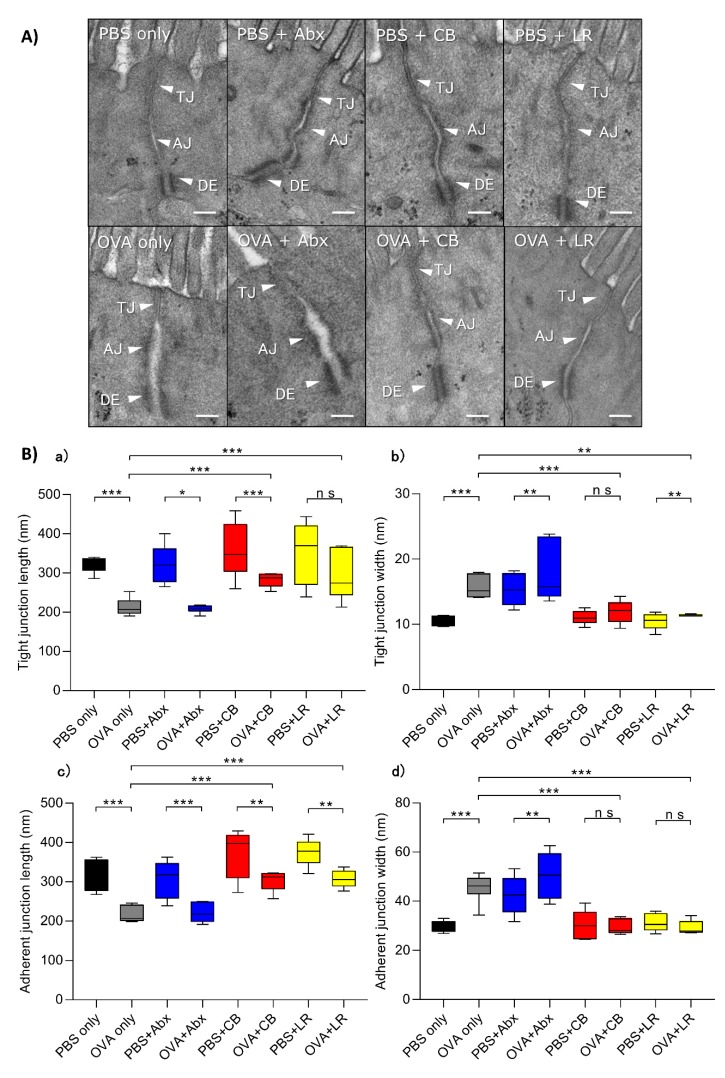
(**A**) Electron microscopy images of epithelial cells. Scale bar indicates 0.5 μm. TJ, tight junction; AJ, adherens junction; DE, desmosome. (**B**) (**a**) Quantitative analysis of TJ length. (**b**) Quantitative analysis of TJ width. (**c**) Quantitative analysis of adherens junction length. (**d**) Quantitative analysis of adherens junction width. Boxes represent the 25th to 75th percentiles; middle bar identifies the median; whiskers show the minimum and maximum (*n* = 5–7 rats per group). * *p* < 0.05, ** *p* < 0.01, *** *p* < 0.001 determined by ANOVA with the Tukey–Kramer method. ns, no significance; Abx, antibiotics; CB, *C. butyricum*; LR, *L. reuteri.*

Compared with the non-sensitized group, damage to the TJs as well as the adherens junction was evident in intestinal villus epithelial cells in the OVA-treated group (OVA only) (Figure 4A). Moreover, in OVA-sensitized rats given antibiotic treatment, the intercellular junctions were shortened and disrupted, and the intercellular spaces were widened. On the other hand, the TJs and adherens junctions in each of the OVA-sensitized and probiotic-treated groups showed almost normal features (Figure 4A). A quantitative analysis of the length and width of TJs revealed significant widening and shortening in the OVA-sensitized group (OVA only) and the OVA-sensitized and antibiotic-treated group (Figure 4Ba,Bb) relative to the control. On the other hand, the OVA sensitization group treated with probiotics did not show TJ widening and shortening. Observations of the adherens junction revealed results similar to those for the TJ (Figure 4Bc,Bd).

### 3.4. mRNA Expression of TJ-Related Molecules in Jejunal Tissue

**Figure 5:**
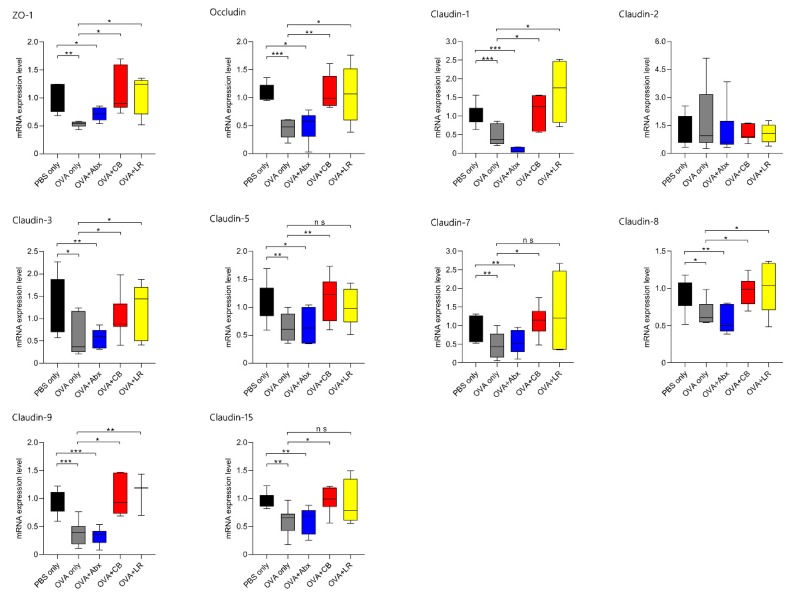
Total RNA was extracted from the jejunal tissue of rats, and the mRNA expression of TJ-related molecules was examined by real time PCR. Data are presented as the fold change in gene expression relative to the control (PBS only). ZO-1; occludin; claudin-1, -2, -3, -5, -7, -8, -9, and -15. Boxes represent the 25th to 75th percentiles; middle bar identifies the median; whiskers show the minimum and maximum (*n* = 5–7 rats per group). * *p* < 0.05, ** *p* < 0.01, *** *p* < 0.001 determined by ANOVA with the Tukey–Kramer method. ns, no significance; Abx, antibiotics; CB, *C. butyricum*; LR, *L. reuteri.*

Expression and localization of key TJ proteins in each group indicated that they were modulated not only by OVA sensitization, but also by the microbiota composition in epithelial cells. As shown in Figure 5, TJ-regulating proteins such as ZO-1 (*p* < 0.01), occludin (*p* < 0.001), claudin-1 (*p* < 0.01), claudin-3 (*p* < 0.05), claudin-5 (*p* < 0.01), claudin-7 (*p* < 0.05), claudin-8 (*p* < 0.01), claudin-9 (*p* < 0.001), and claudin-15 (*p* < 0.01) were significantly down-regulated in OVA-sensitized rats (OVA only) relative to the PBS only controls. However, expression of claudin-2 was not significantly increased. 

In the OVA-sensitized and antibiotic-treated rats, ZO-1 (*p* < 0.05), occludin (*p* < 0.01), claudin-1 (*p* < 0.001), claudin-3 (*p* < 0.01), claudin-5 (*p* < 0.05), claudin-7 (*p* < 0.01), claudin-8 (*p* < 0.01), claudin-9 (*p* < 0.001), and claudin-15 (*p* < 0.01) were also down-regulated relative to the PBS only group. On the other hand, in each of the groups treated with probiotics (*C. butyricum*, and *L. reuteri*), the expression levels of TJ proteins (ZO-1; occludin; claudin-1, -3, -5, -7, -8, -9, and -15) were up-regulated relative to the OVA only group (except for claudin-5, -7, and -15 after administration of *L. reuteri* to OVA-sensitized rats). 

**Figure 6:**
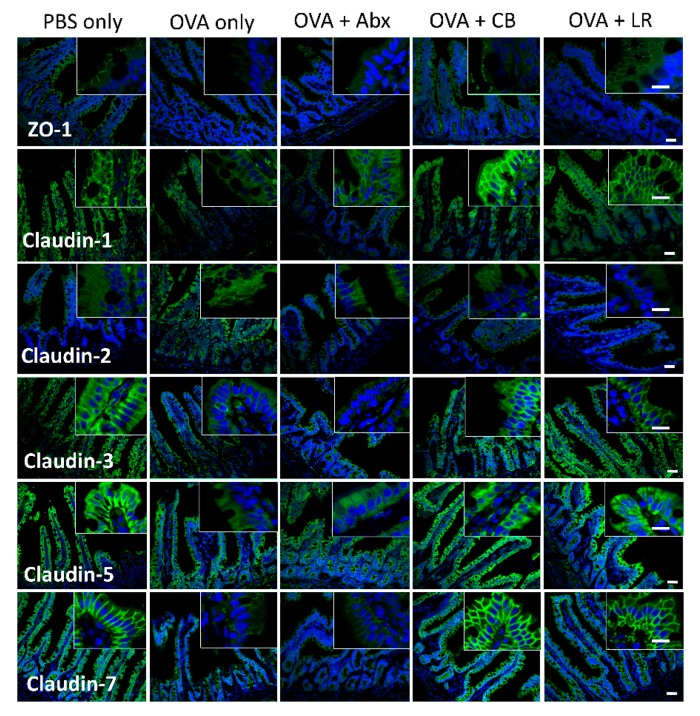
Representative confocal images of TJ-related proteins. Cryosections of the jejunal tissue were immunolabeled for ZO-1 and claudin-1, -2, -3, -5, and -7 (in green). Nuclei were visualized with DAPI (in blue). Size bars, 50 μm (small) and 20 μm (large). Abx, antibiotics; CB, *C. butyricum*; LR, *L. reuteri*.

As shown in Figure 6, immunofluorescence staining for intestinal TJ proteins such as ZO-1 and claudin-1, -2, -3, -5, and -7 in each group indicated that not only OVA sensitization, but also microbiota composition appeared to be related to the localization of those proteins in epithelial cells. In the PBS only (control) group, ZO-1 was expressed in the apical membrane of epithelial cells. In the OVA-sensitized (OVA only) group and OVA-sensitized groups treated with antibiotics, ZO-1 expression was lost in the apical membrane. Claudin-1, -3, -5, and -7 were stained in the apicolateral area in the PBS only (control) groups. In the OVA only group and OVA-sensitized groups treated with antibiotics, the expression of these proteins was weaker and located mainly in the basolateral membrane of the villi. In contrast, in each of the probiotic-treated groups, the expression of claudin proteins was mild and their localizations were similar to those in the controls (PBS only).

### 3.5. Gut Microbiota Is Associated with Expression of Th2 and Inflammatory Cytokines

**Figure 7:**
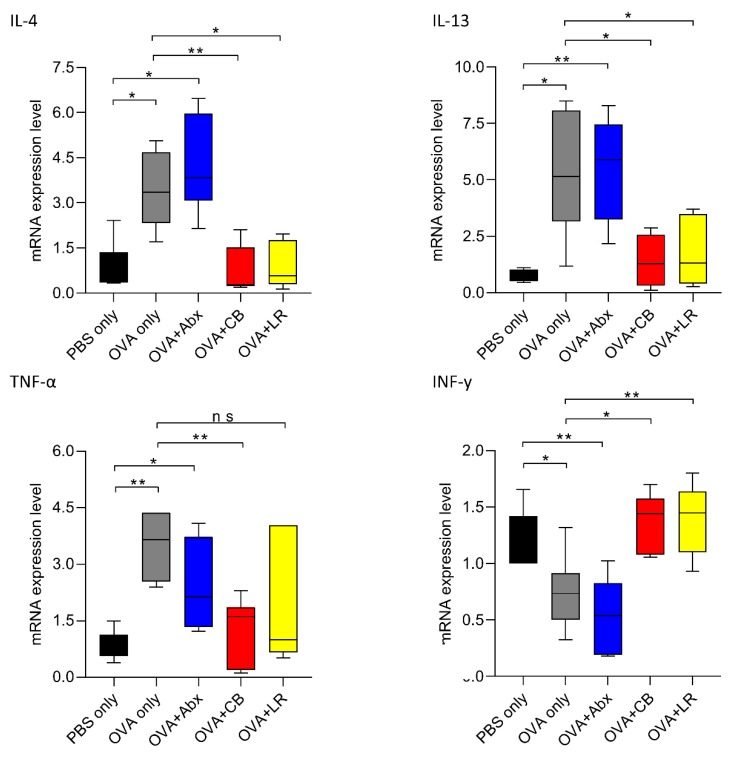
Total RNA was extracted from the jejunal tissue of rats, and the mRNA expression of Th2 and inflammatory cytokine were examined by real time PCR. Data are presented as the fold change in gene expression relative to the control (PBS only). Interleukin (IL)-4, IL-13, tumor necrosis factor (TNF)-α, and interferon-gamma (INF-γ). Each value is represented as the mean ± SEM (*n* = 5–7 rats per group). Boxes represent the 25th to 75th percentiles; middle bar identifies the median; whiskers show the minimum and maximum (*n* = 5–7 rats per group). * *p* < 0.05, ** *p* < 0.01 determined by ANOVA with the Tukey–Kramer method. ns, no significance; Abx, antibiotics; CB, *C. butyricum*; LR, *L. reuteri.*

We investigated the levels of cytokines associated with allergic reaction and inflammation. This revealed that levels of expression of mRNAs for interleukin (IL)-4, IL-13, and tumor necrosis factor (TNF)-α in the jejunum were increased in the OVA-sensitized rats (OVA only) and the OVA-sensitized rats given antibiotics, respectively, but not in those given probiotics (except for TNF-α after administration of *L. reuteri*). The results for interferon-gamma (INF-γ) were opposite to those for the above cytokines (Figure 7). 

### 3.6. Influence of OVA Sensitization on Composition of the Fecal Microbiota

**Figure 8:**
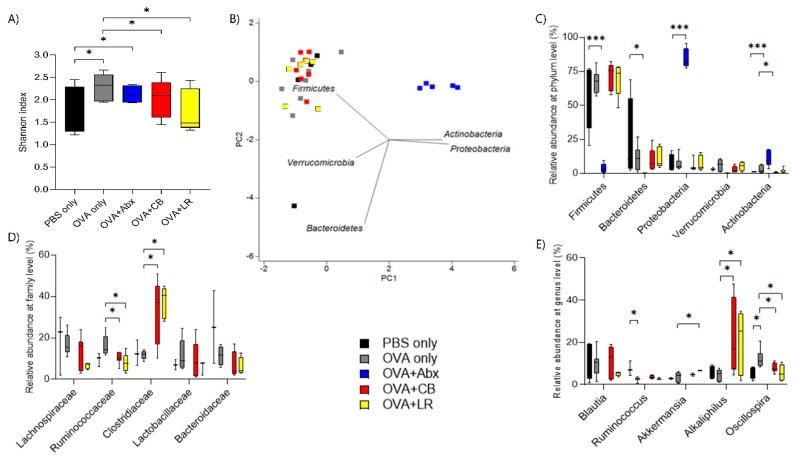
(**A**) Diversity profiles of the gut microbiota. (**B**) Principal component analysis at the phylum-level in fecal contents. (**C**) Phylum-level taxonomic distributions of the microbial communities. (**D**) Composition of the gut microbiota at the family level. Lachnospiraceae, Ruminococcaceae, Clostridiaceae, and Bacteroidaceae were not detected in the antibiotics treated groups. (**E**) Composition of the gut microbiota at the genus level. *Blautia*, *Ruminococcus*, *Akkermansia*, *Alkaliphilus*, and *Oscillospira* were not detected in the antibiotics treated groups. Boxes represent the 25th to 75th percentiles; middle bar identifies the median; whiskers show the minimum and maximum (*n* = 5–7 rats per group). * *p* < 0.05, *** *p* < 0.001 determined by permutational multivariate analysis of variance (PERMANOVA). ns, no significance; Abx, antibiotics; CB, *C. butyricum*; LR, *L. reuteri*.

Analysis of the overall bacterial community structure demonstrated that rats treated with *C. butyricum* and *L. reuteri* had a significantly less diverse community than OVA-sensitized rats without probiotic treatment (OVA only) (Shannon’s index, OVA = 2.3 ± 0.2 vs. OVA + *C. butyricum* = 1.8 ± 0.3; OVA + *L. reuteri* = 1.7 ± 0.4; Figure 8A).

From the comparison of relative abundance at the phylum level, sequences specific for Proteobacteria and Actinobacteria were evident in the antibiotic-treated groups (Figure 8B,C). The family Clostridiaceae was significantly increased and Ruminococcaceae was decreased in both of the groups administered probiotics (*C. butyricum* and *L. reuteri*) relative to the OVA-sensitization group (OVA only) (Figure 8D). In the genus level analysis, *Ruminococcus* was decreased in the OVA only group relative to the PBS only group (control), and recovered by probiotics treatment. *Akkermansia* in the OVA sensitized with *L. reuteri* group, and *Alkaliphilus* in both of the groups administered probiotics, were increased compared with the OVA only group, respectively. *Oscillospira* was significantly enriched in the OVA only group, but reduced by administration of *C. butyricum* or *L. reuteri* (Figure 8E). 

### 3.7. Summary of the Results by Group

#### 3.7.1. OVA Sensitization

Gut permeability, the serum-specific OVA-IgE level, intestinal mucosal inflammation score, and mucosal eosinophilic infiltration were increased. TJ structures were disrupted, and expression of TJ proteins was mislocalized (ZO-1 and claudin-1, -3, -5, and 7) and down-regulated (ZO-1; occludin; claudin-1, -3, -5, -7, -8, -9, and -15). mRNA expression of IL-4, IL-13, and TNF-α were increased in the jejunum. The bacterial community structure was more diverse, and the genus *Oscillospira* was enriched.

#### 3.7.2. OVA Sensitization with Antibiotics 

Gut permeability was increased, and a higher level of serum-specific OVA-IgE, severe inflammation of the mucosae, as well as TJ structural damage were demonstrated by antibiotic treatment in the OVA sensitization group. Expression of TJ proteins (ZO-1; occludin; claudin-1, -3, -5, -7- 8, -9, and 15) was markedly down-regulated, and low expression of the proteins (ZO-1 and claudin-1, -3, -5, -and 7) was detected. mRNA expression of IL-4, IL-13, and TNF-α was increased in the jejunum. The composition of gut microbiota was quite different from that of the commensal bacterial flora.

#### 3.7.3. OVA Sensitization with Probiotics 

Neither gut permeability nor the serum-specific OVA-IgE level were increased, and allergic mucosal inflammation was mild. TJ proteins were not down-regulated and showed a similar intensity of immunofluorescence staining to that in the controls. mRNA expression of INF-γ was increased, but that of IL-4, IL-13, and TNF-α was decreased in the jejunum. The gut microbiota demonstrated less diversity in comparison with rats subjected to OVA sensitization (OVA only), and enrichment of Clostridiaceae. *Akkermansia* and *Alkaliphilus* were significantly increased by administration of *L. reuteri*, and the latter was also significantly increased by *C. butyricum* administration, respectively.

## 4. Discussion

In the present study, using juvenile–young adult rats with food sensitization as a model, we found that not only antigen (OVA) sensitization, but also antibiotic treatment induced leaky gut, causing allergen absorption and food sensitization, presumably through significant expression of key TJ proteins. Moreover, administration of probiotics prevented this increase in intestinal permeability, presumably through an increase in Clostridiaceae, as well as a significant influence on the expression of TJ proteins. 

One of the functions of the epithelial barrier is to prevent macromolecular antigens and other harmful substances from being absorbed [23,24]. It is reported that intestinal permeability may increase when the intestinal mucosal barrier function is damaged during allergy, and that such epithelial barrier dysfunction leads to excessive transport of macromolecular substances, which are absorbed into the deep tissues and have the potential to induce antigen-related intestinal inflammation or hypersensitivity [25,26,27,28]. The results of our lactulose/mannitol assay showed that intestinal permeability was increased after OVA sensitization, and this was further confirmed by electron microscopy (shortened and widened intercellular TJs of intestinal villus epithelial cells). These changes in OVA-sensitized rats were not prevented by administration of antibiotics, but were prevented by treatment with probiotics, as further confirmed by electron microscopy. These findings are in line with a previous report indicating that TJ and adherens junction microstructure is markedly disrupted and widened after OVA sensitization and treatment with antibiotics [29].

TJs play an important role in the maintenance of intestinal permeability, which is considered to determine selective cellular absorption [30,31]. TJs comprise multiple proteins forming a functional complex, and the major function of most TJ proteins is to maintain the integrity of the epithelial barrier. Transmembrane proteins, such as occludin, claudins, and junctional adhesion molecules, are structural proteins arranged in a linear manner, whereas the cytoplasmic adhesion proteins, ZO-1, -2, and -3, form a supporting cytoskeletal structure [32]. These proteins play a pivotal role in the regulation of TJ permeability and intestinal barrier function [33,34,35,36,37,38]. Our real-time PCR study showed that OVA down-regulated the expression of ZO-1; occludin; and claudin-1, -3, -5, -7, -8, -9, and -15. These findings are in line with a previous similar study of OVA-sensitized rats, which demonstrated significant down-regulation of the TJ-mediating proteins ZO-1 and claudin-8 and -15 [19]. These results suggest that OVA induces damage to the intestinal barrier, and that TJ permeability is related to the expression and regulation of these proteins. Although a previous report has indicated that elevated expression of claudin-2 in epithelial cells plays an important role in epithelial barrier dysfunction as well as the pathogenesis of intestinal antigen-specific hypersensitivity [39], we found no significant increase of claudin-2 expression in the present study. This may have been partly owing to the experimental design, as another previous report that adopted a similar experimental design also demonstrated no significant differences in the claudin-2 level between an antigen sensitization group and a control group [19]. On the other hand, treatment with *C. butyricum* and *L. reuteri* up-regulated the expression of ZO-1; occludin; and claudin-1, -3, -5, -7, -8, -9, and -15. Similar beneficial effects of probiotics on the association between intestinal permeability and TJ protein expression have been reported. In Bet v1 pollen-sensitized mice, a mixture of *Lactobacillus* strains up-regulated the expression of occludin and the TJ molecule ZO-1, and improved the function of the gut epithelial barrier [29]. In rats treated with dextran sulfate sodium, which has been widely used as a model for inducing acute and chronic colitis, a probiotic mixture of strain VSL#3 was shown to protect against increased intestinal permeability by up-regulating of expression of occludin, ZO-1, and claudins 1–5 [40].

Using an OVA-sensitized rat model, we further detected downregulation of the Th2-associated cytokines IL-4 and IL-13, and upregulation of the Th1-associated cytokine INF-γ in the groups administered probiotics, suggesting that probiotics induced an immunoregulatory response. These findings are consistent with previous studies showing that probiotic administration or supplementation exerted beneficial effects by improvement of the Th1/Th2 balance in a mouse model of food allergy [13,19,29]. Regarding the mechanism of tolerance acquisition in infancy, it involves a complex combination of physiological and immunological regulation. One of the factors involved is food antigen degradation in the lumen as the digestive function develops. Others include prevention of the absorption of high-antigenic substances through the development of gut barrier function and secretory IgA production. Organization of the gut epithelium makes it a tight, efficient barrier with filtering properties against the entry of allergens. Secretory IgA-based immune complexes promote the induction of non-inflammatory cytokines, ensuring low reactivity against transported antigens. In addition, regulatory T cells and clonal deletion of antigen-specific T cells, as well as anergy induction, are crucial factors in tolerance [41]. Our study suggests that probiotics could be advantageous in terms of tolerance because neither gut permeability nor the serum-specific IgE level were increased, and expression of mRNA for IL-4, IL-13, and TNF-α was decreased in the jejunum. 

The microbiota of OVA-sensitized rats was significantly more diverse than that of each of the probiotic treatment groups and control (PBS only) group. This is compatible with previous clinical reports that demonstrated that the microbiota of children with food allergy was significantly more diverse than that of healthy controls [42,43,44]. Clostridiaceae were also significantly enriched by treatment with *C. butyricum* and *L. reuteri*. It was possible to rule out any contamination because the rats were housed in individual ventilation cages to keep them separated from the other rats and any possible exposures, including that via air. Furthermore, we paid careful attention to all technical procedures, agents, and equipment (probiotic solutions or gavage tubes were kept in different places) to avoid contamination.

It is reported that a combination of specific immunotherapy with *C. butyricum* significantly enforces the therapeutic effect against food allergen-related inflammation in mouse intestine [12]. Stefka et al. reported that *Clostridium* regulates innate lymphoid cell function to alter gut epithelial permeability and reduce allergen uptake into the systemic circulation [10]. *C. butyricum* can produce butyrate, which is not only the main energy source for enterocyte regeneration [45], but also an important immunomodulatory molecule in the intestine. Butyrate can stimulate the rearrangement of TJ proteins of epithelial cells and improve gut barrier integrity [46].

*Akkermansia* and *Alkaliphilus* were significantly increased in the OVA sensitized with *L. reuteri* administration group, and the latter was also significantly increased in the OVA sensitized with *C. butyricum* administration group in comparison with the OVA only group, respectively. Recently, a reduced *Akkermansia muciniphila* level in the gut microbiota of children with allergic asthma has been reported [47]. Another study demonstrated that *A. muciniphila* significantly increased the expression of occludin and claudin-4. Both *A. muciniphila* and extracellular vesicles increased the expression of ZO-2 in the cell line. The researchers concluded that *A. muciniphila* and its extracellular vesicles might both increase the integrity of the intestinal barrier and reduce inflammation [48]. *A. muciniphila* is a mucus-colonizing member of the gut microbiota that has evolved to specialize in the degradation and utilization of host mucus. Mucus degradation and fermentation by *A. muciniphila* are known to result in the production of acetate [49]. *L. reuteri* also produces lactate and acetate, which become directly available to coexisting butyrogenic bacteria, which normally produce butyrate through carbohydrate fermentation or amino acids degradation pathways within the same mucosal niche. Aside from stimulation of the rearrangement of TJ proteins in epithelial cells and improvement of gut barrier integrity, butyrate can stimulate mucus production from epithelial cells [46]. Therefore, one of the reasons for the increase of *Akkermansia* revealed by the fecal microbiota analysis of the *L. reuteri* administration group could have been thickening of the mucus layer, leading to enhancement of gut barrier integrity in a way. There is a “coexistence cycle” associated with *Akkermansia*, so to speak, and the acceleration of mucus production by butyrate may lead to an increase in the number of *Akkermansia*. Similar effects are expected for *C. butyricum*, which produces butyrate, although the fecal microbiota analysis revealed no significant differences. On another note, there is little information about gut *Alkaliphilus*. *Alkaliphilus transvaalensis* is reportedly connected to chitin degradation with production of high short chain fatty acids (SCFAs; acetate, propionate, and butyrate) [50]. Thus, although speculative, *Alkaliphilus* may be integrated into the gut microbiota ecosystem where many microorganism networks are formed, and produce SCFAs. 

In our present study, *Oscillospira* was significantly enriched in the OVA only group, and reduced in both the *C. butyricum*-treated and *L. reuteri*-treated OVA groups. A previous report has revealed that dietary intervention with extensively hydrolyzed casein formula supplemented with *L. rhamnosus* GG accelerates tolerance acquisition in infants with cow’s milk allergy. Fecal samples from infants with cow’s milk allergy demonstrated that *Oscillospira* was reduced in infants that became tolerant, but significantly enriched in those that remained allergic [44]. Therefore, from the viewpoint of *Oscillospira*, it seemed that both *C. butyricum* and *L. reuteri* played a beneficial role in our study.

At the species level, the probiotics we administered were not detected at all by a fecal microbiota analysis (data not shown). Therefore, it is considered that these probiotics did not colonize the gut. They might have passed through the gut within one day and fallen below the detection sensitivity limit. A previous study has also reported that administered *C. butyricum* grew in, but did not colonize the rat intestine because intestinal *C. butyricum* cells disappeared (ELISA) three days after administration [51]. Further investigation will be needed in order to clarify why none of the administered probiotics were detected.

We found that OVA sensitization alone changed the composition of gut microbiota in comparison with controls, in terms of the relative abundance of bacterial phyla. Similarly, Andreassen et al. recently reported that allergen immunization in a food allergy model induced profound changes in the composition of the gut microbiome [52]. This is considered to indicate that the gut mucosal immune system can affect the composition of gut commensal bacteria. In other words, there is a bidirectional interaction between gut microbiota formation and the mucosal immune system. The gut microbiota signatures could have been affected by OVA sensitization through complex ecosystem effects.

Because the jejunum, where most food absorption occurs in the digestive tract, plays an important role in epithelial barrier function in food allergy, we focused on the jejunum in the present study. However, microbiota composition and immune cell composition vary throughout the intestine, and these regional variations will influence the interactions between them. Therefore, further investigation of different segments of the gastrointestinal tract (ileum, colon, and so on) is needed. We acknowledge that the sample size used in this study was small, and this is the major limitation of our study. Furthermore, despite the use of inbred rodents and standardized maintenance, handling, and exposure to the animals in the same groups, there is considerable inter-individual variation in the immune responses. A previous study demonstrated that some of the immunized experimental animals appeared to be non-responders or low responders to immunization owing to inter-individual variation, even though the animals were brought up in the same environment and treated in the same manner [52]. Therefore, our results might have been affected by non-immune response bias. 

A recent ground-breaking clinical trial demonstrated that the combination of probiotics (*L. rhamnosus*) and peanut oral immunotherapy provided a long-lasting clinical benefit and persistent suppression of the allergic immune response in children with peanut allergy [53]. In that study, the microbial composition of stool samples was not analyzed in order to examine the effects of the combination therapy, and the synergistic action of probiotics with oral immunotherapy was not assessed; however, the findings suggest that probiotics may be able to enhance the tolerance-inducing capacity of oral immunotherapy. Further basic experiments and clinical trials are needed in order to address the important and as-yet-unanswered question of whether or not probiotics provide a significant benefit for the prevention or treatment of food allergy.

## 5. Conclusions

In conclusion, OVA sensitization is able to induce an increase of intestinal permeability by influencing TJ protein regulation. Gut microbiota plays a role in regulating epithelial barrier function and probiotics may help to prevent food sensitization through up-regulation of tight junction proteins.

## Figures and Tables

**Table 1:** Primers used in real time-PCR analysis. ZO, zonula occludens.

Primer	Sequence (5′-3′)
ZO-1 F	5′-ACCCACGAAGTTATGAGCA AG-3′
ZO-1 R	5′-AGACTGTGGTTTCATTGC TGG-3′
Occludin F	5′-ATTCCTCTGACCTTGTC CGTG-3′
Occludin R	5′-CCTGTCGTGTAGTCG GTTTCA-3′
Claudin-1 F	5′-AGGTCTGGCGACATTAGTGG-3′
Claudin-1 R	5′-GAAGGTGTTGGCTTGGGATA-3′
Claudin-2 F	5′-ATTCCTCTGACCTTGTC CGTG-3′
Claudin-2 R	5′-AGCCAACCGCCGTCAC AATG-3′
Claudin-3 F	5′-GCACCCACCAAGATCCTCTA-3′
Claudin-3 R	5′-AGGCTGTCTGTCCTCTTCCA-3′
Claudin-5 F	5′-CACAGAGAGGGGTCGTTGAT-3′
Claudin-5 F	5′-CAGCTGCCCTTTCAGGTTAG-3′
Claudin-7 F	5′-ATGCTCCTGGATTGGTCATC-3′
Claudin-7 F	5′-GTCCCCAGCTCACACGTATT-3′
Claudin-8 F	5′-TGTCGTGTTTGAGAA CCGCTGGG-3′
Claudin-8 R	5′-ACGGACGCAG CACACATCAGTC-3′
Claudin-9 F	5′-TTCCACTGGCCTTG AACTCCTCG-3′
Claudin-9 R	5′-GCTGTTGCCAA TGAAGGCGGT-3′
Claudin-15 F	5′-AACTGCTGGGACTT CCCGTCCAT-3′
Claudin-15 R	5′-TCGATGTTGCCC ACGTTGGTGC-3′
IL-4 F	5′- TCCTTACGGCAACAAGGAAC-3′
IL-4 R	5′-CAGTGTTGTGAGCGTGGACT-3′
IL-13 F	5′-CTGAGCAACATCACACAAGACC-3′
IL-13 R	5′-TTGCAACTGGAGATGTTGGTCAG-3′
TNF-α F	5′-ACTCCCAGAAAAGCAAGCAA-3′
TNF-α R	5′-CGAGCAGGAATGAGAAGAGG-3′
INF-γ F	5′- CAATAGTTGTCCCGGCACTT-3′
INF-γ R	5′- GGTGCTTCTAGGCTGTCTGG-3′
β-actin F	5′-GTCTCACCACTGGCA TTGTG-3′
β-actin R	5′-TCTCAGCTGTGGTGGT GAAG-3′

F: Forward; R: Reverse.
